# The Effects of Legume-Cereal Intercropping on the Symbiotically Fixed N_2_ in Soybean, N Accumulation, and C Allocation

**DOI:** 10.3390/plants14071009

**Published:** 2025-03-24

**Authors:** Monika Toleikiene, Raminta Skipityte, Ruta Bariseviciute, Juliana Trindade Martins, Jim Rasmussen

**Affiliations:** 1 Institute of Agriculture, Lithuanian Research Centre for Agriculture and Forestry, Instituto al, 1, LT-58344 Akademija, Lithuania; 2 Center for Physical Sciences and Technology, Savanorių Av. 231, LT-02300 Vilnius, Lithuania; raminta.skipityte@ftmc.lt (R.S.); ruta.bariseviciute@ftmc.lt (R.B.); 3 Department of Agroecology, Aarhus University, Blichers Allé 20, DK-8830 Tjele, Denmark; jtm@agro.au.dk (J.T.M.); jim.rasmussen@agro.au.dk (J.R.)

**Keywords:** soybean, spring wheat, ^15^N-labelling, ^13^C-allocation, C and N content

## Abstract

Intercropping soybean and wheat can enhance soil fertility through increased nitrogen fixation, optimize resource use, and boost overall crop productivity, thereby promoting sustainable agricultural practices. Thus, this research examines nitrogen accumulation and carbon allocation in the intercrops of soybean and spring wheat, as well as the nitrogen fixation in soybean using the ^15^N isotope dilution method and ^13^C-CO_2_ pulse labeling. Soybean and spring wheat were grown as monocultures and mixtures in different densities, containing 4 or 8 plants of wheat, either 1 or 3 soybean plants, or a mixture of both. The intercropping had a significant impact on soybean atmospheric nitrogen fixation. When grown in mixtures with wheat, soybean accumulated more than twice as much atmospheric nitrogen in the roots; however, the effect on total accumulated N per plant was rather negative and plant densities dependent. Growing mixtures at low densities of soybean and high densities of wheat had a better effect on the total nitrogen content of plants. Overall, intercropping caused a significant redistribution of carbon and nitrogen in plants. Carbon allocation was influenced in soybeans but not in wheat grown in monocultures and in mixtures. Intercropping also positively influenced carbon accumulation, with the increase in carbon density being more pronounced in the roots than in the shoots for both species.

## 1. Introduction

Legume (*Fabaceae*) form a symbiotic association with specific rhizobia bacteria in the soil to fix atmospheric nitrogen, which is perceived as a strategy to reduce the use of inorganic fertilizers and the N import from livestock [1]. However, the N fixation varies considerably depending on legume species, as well as the local soil and climatic conditions [2]. Across varying pedoclimatic conditions, biological N fixation (BNF) for different legume species ranges from a few to several hundred kg ha^−1^ annually [3]. Rochester et al. [4] showed that soybean can fix 453–488 kg N ha^−1^ per year. After the seed harvest, it can contribute to the soil nitrogen pool by 155–280 kg fixed N ha^−1^. Therefore, to optimize the use of legumes in our cropping systems, we need to understand how management can enhance the N use from BNF.

In soybean production, effective rhizobial symbiosis is crucial for soybean production and overall soil fertility [5]. In soils without a previous history of soybean cultivation, it is necessary to inoculate with the *Bradyrhizobium japonicum* strains in order to secure nodulation and sustain nitrogen fixing efficiency [6]. Effective inoculation with *Bradyrhizobium* strains increases grain yield, biomass, protein content, and yield [7]. In regions without prior history of soybean cropping, like Northern Europe, where soybean yields may be challenged in years with unfavorable weather conditions, intercropping of soybean with spring cereals could be an option to create more favorable micro-climates for the soybean plants and a means to secure yields for the farmers. The growing of spring cereals together with grain legumes has a positive effect on soil fertility [8], contributes to long-term N immobilization [9], enhances the efficiency of resource use such as light, heat, physical space, water, and soil nutrients [10,11,12], benefits the companion/subsequent crops [13,14], and reduces yield instability as compared to sole grain legumes [15]. Globally, cereal-soybean intercropping is the most common [16], while in Europe, mixtures such as pea-wheat [17] or pea-oat [18] dominate. Although several studies show a positive N effect on aboveground yields and N contents [19,20,21], we still lack studies on the interaction between soybean and companion cereals.

In intercrops, there are distinct positive and negative relationships between the companion plants. Interspecific or intraspecific competition arises in response to the availability of resources within the agroecosystem. The sowing rate and density of the crops influence the degree of resource complementarity and therefore affect the total yield [22]. Plant density and sowing proportions significantly affect the interspecies dynamics of intercrops [23], although the optimal sowing rate and plant density observed in one location may not be applicable to others due to the meteorological variations and the different soil properties [23]. The interspecies dynamics affect the internal cycling of primary and specialized metabolites and the C and N cycling within and between intercropped species [24,25]. Intercropping typically leads the legumes to increase reliance on BNF [26]. Corre-Hellou et al. [27] showed that N derived from the atmosphere (%Ndfa) in the aboveground biomass of peas increased by 20% in the mixture with cereals as compared to sole cropping, and Kumar and Goh [28] found that more than 80% of the N was derived from BNF. The legume biomass production also affects the ratio of legume nitrogen derived from N_2_-fixation [29], which in intercropping systems may be affected by companion species competition for above and below-ground resources. Grain legumes cultivated as sole crops often fix a higher amount of the total N than intercrops due to a greater biomass accumulation [30], but we need studies on the effect of intercropping C allocation to shoot and root in companion species.

Our study investigates the effects of legume-cereal intercropping on symbiotically fixed nitrogen in soybeans, nitrogen accumulation, and carbon allocation within soybean-wheat intercrops. Although legume-cereal intercropping is widely recognized for its benefits in enhancing nitrogen availability and resource use efficiency, the extent to which intercropping influences nitrogen fixation and carbon allocation in legumes remains unclear. To address this gap, we examined plant interactions at varying densities under controlled conditions. We specifically hypothesized that intercropping would enhance atmospheric nitrogen fixation and belowground carbon allocation in soybean compared to monocultures and that competitive interactions between species would increase soil-derived nitrogen uptake in wheat.

## 2. Results

### 2.1. Intercropping Effect on Biological Parameters of Plants

As described in the methods section, each pot contained either 4 or 8 wheat plants, 1 or 3 soybean plants, or a mixture of both. The treatments were as follows: monocultures included (1) non-inoculated soybean as a control, (2) low-density soybean (1 plant), (3) high-density soybean (3 plants), (4) low-density wheat (4 plants), and (5) high-density wheat (8 plants). Mixed treatments included (6) high-density soybean with low-density wheat (3 + 4 plants, respectively), (7) low-density soybean with low-density wheat (1 + 4 plants, respectively), and (8) low-density soybean with high-density wheat (1 + 8 plants, respectively). Plant height, biomass, and C:N ratios in shoots and roots of soybean and spring wheat, cultivated as monocultures or intercrops at various plant densities, are presented in Table 1.

The data show that neither soybean height (which ranged from 27.2 to 34.0 cm on average) nor shoot biomass (which ranged from 1.52 g of plant^−1^ DM to 2.68 g plant^−1^ DM, and C:N varied within 13.4–17.0 on average) was significantly affected by intercropping with spring wheat. Root weight was slightly (from 0.66–0.73 g plant^−1^ DM to 0.34–0.46 g plant^−1^ DM on average) but not significantly lower in soybeans cultivated in mixtures. However, the change in the C:N ratio in roots (from 10.8–12.3 to 16.0–17.9 on average) indicates that intercropping caused a significant redistribution of carbon and nitrogen in plants.

Wheat, on the other hand, was more sensitive to plant density than to intercropping, as a significant increase in root C:N values (from 19.8 to 20.8–24.9) was observed when cultivated in mixtures or high-density monoculture compared to growth in low-density monoculture. Plant height (50.3 cm in low-density monoculture and 43.2–45.0 g plant^−1^ DM in high-density systems on average) was also reduced in dense systems, as was root biomass (0.49 g plant^−1^ DM in low-density monoculture compared to 0.22–0.34 g plant^−1^ DM in high-density systems on average).

### 2.2. Intercropping Effect on N Content, NDFA and C Allocation

Data of nitrogen and carbon content, as well as %Ndfa and ^13^C isotope allocation in the shoots and roots of soybean and spring wheat, cultivated as monocultures and intercrops at various plant densities, are presented in Table 2.

Nitrogen content varied from 2.63 to 3.14% on average in soybean shoots, and this variation was not statistically significant; meanwhile, the variation in its roots was significantly higher in monocultures (3.12–3.59% on average) compared to the mixtures (2.39–2.64% on average). The average nitrogen content in spring wheat was less variable in both shoots (2.19–2.55%) and roots (1.49–1.88%) compared to soybean, but significantly lower in shoots in monocultures to compare with overall mixtures.

Nitrogen fixation expressed as nitrogen derived from the atmosphere (%Ndfa) was significantly affected by intercropping during the flowering stage of soybean. %Ndfa increased significantly in soybean roots from 6.0% in monocultures to 12.8–15.3% in mixtures during the flowering period. In shoot tissue, the average %Ndfa varied from 2.5 to 5.1% in the monocultures and from 2.9 to 9.2% in the mixtures. The highest but not significant concentration of atmospheric nitrogen was observed in soybeans cultivated in mixtures with high-density spring wheat.

The average percentage of C in the shoots (42.2–43.7%) did not differ statistically significantly between soybeans in either low or high densities in monocultures and mixtures; however, it was higher (44.5%) in mixtures in low densities of both soybean and spring wheat plants. The intercropping increased C concentration in soybean root tissues from 38.4–39.0% to 40.8–42.2% on average. The intercropping also significantly increased the carbon content in the wheat roots (from 30.9–32.7% to 38.4–39%). The wheat shoots had a slightly but not significantly higher C content (43.2–43.9%) in the mixtures than those grown in monocultures (42.5–42.6%), apart from those grown in mixtures with low soybean and wheat densities (45.4%). In general, the impact of intercropping on carbon densities is more pronounced in the roots than in the shoots for both species.

Intercropping does not alter ^13^C allocation from shoots to roots in spring wheat; however, it reduced it by 17% (from 40.9–42.2% to 33.2–35.8% on average) in soybean.

### 2.3. Carbon and Nitrogen Content in Individual Plants

**Figure 1:**
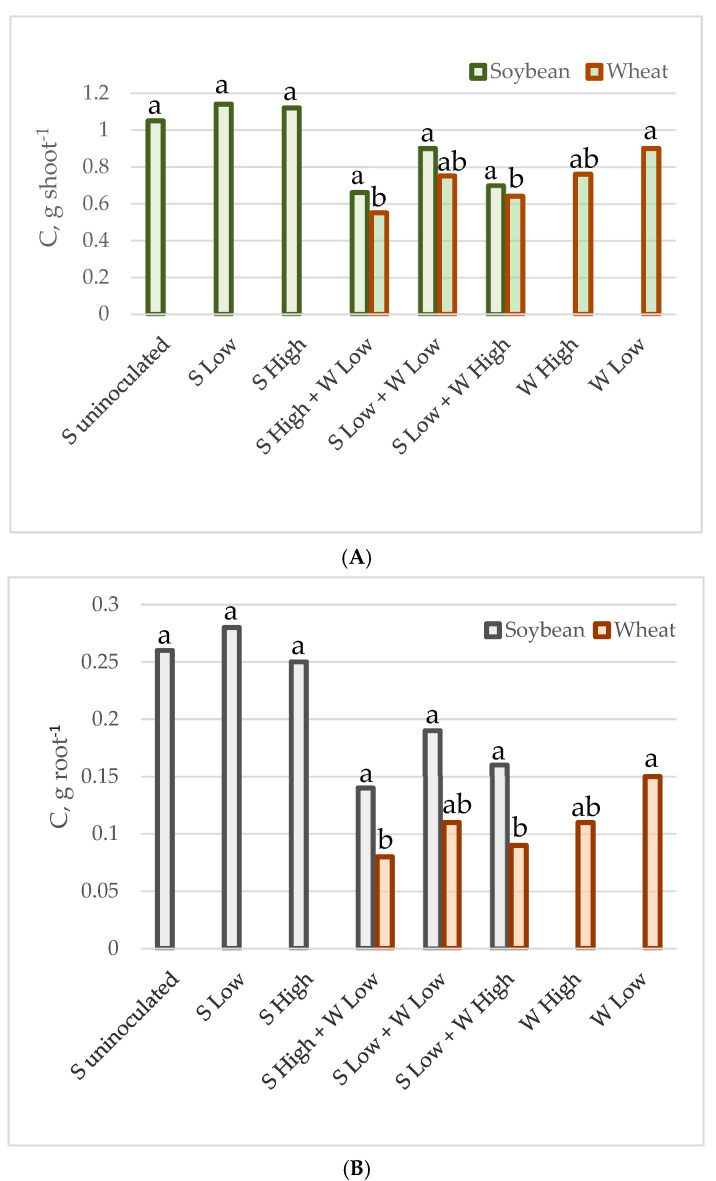
C accumulated by individual plant shoot (**A**) and root (**B**) in g.

**Figure 2:**
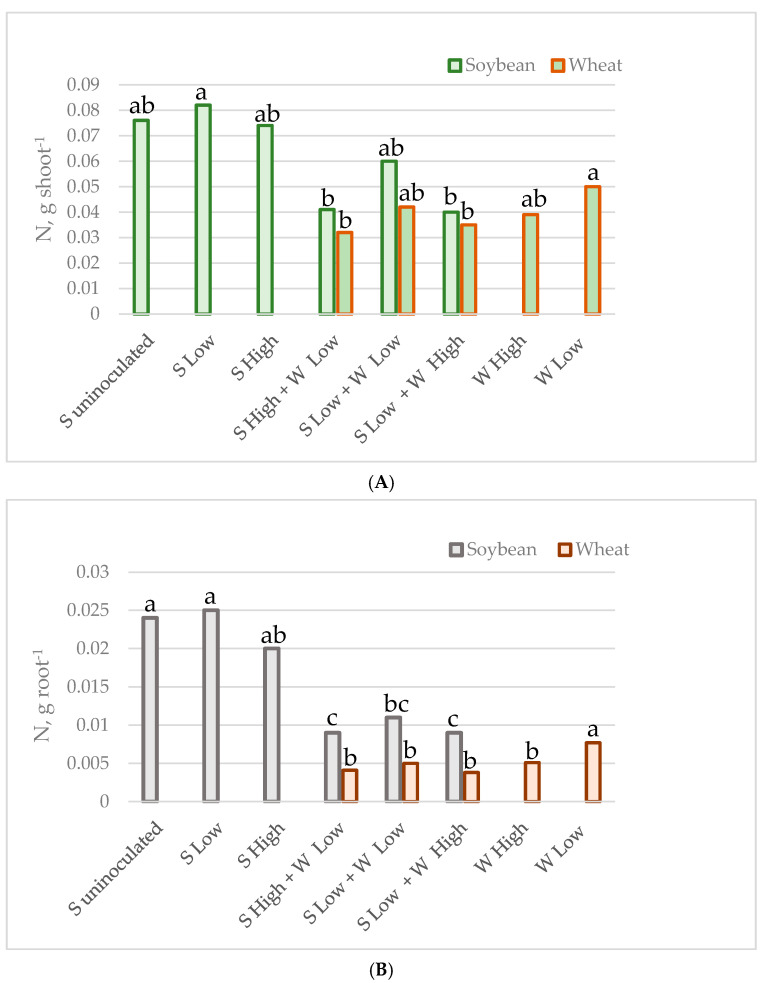
N accumulated by individual plant shoot (**A**) and root (**B**) in g.

The mean carbon and nitrogen content in the shoots and roots of individual plants of soybean and spring wheat, grown as monocultures or intercrops at various plant densities, is presented in Table 3 and Figure 1 and Figure 2. In general, the carbon and nitrogen content was higher in soybeans than in wheat. The intercropping reduced nitrogen content in both the root and the shoot of soybean. The nitrogen content was reduced by 39% in the soybean’s shoot (from 0.074–0.082 g to 0.041–0.060 g on average), while in the roots the reduction reached 58% (from 0.020–0.025 g to 0.009–0.011 g on average). The intercropping did not significantly alter the carbon content in the shoots and roots; however, it was slightly lower in the mixtures (from 1.05–1.12 g to 0.70–0.90 g, and from 0.25–0.28 g to 0.14–0.19 g on average). Wheat was more affected by the density of the plants in the pot than by the presence of soybean, as the shoots and roots had a higher concentration of both elements in the crops from the experiment with low density in monocultures (0.050 g and 0.0077 g of nitrogen, as well as 0.9 g and 0.15 g of carbon in shoots and roots, respectively). The C and N content in wheat from the other cases was not statistically significantly different. The nitrogen content ranged from 0.032–0.042 g in the shoots and from 0.0038–0.0051 g in the roots, while the range of carbon values was 0.55–0.76 g in the shoots and 0.08–0.11 g in the roots on average.

### 2.4. Accumulated Total Carbon and Nitrogen in Pots

**Figure 3:**
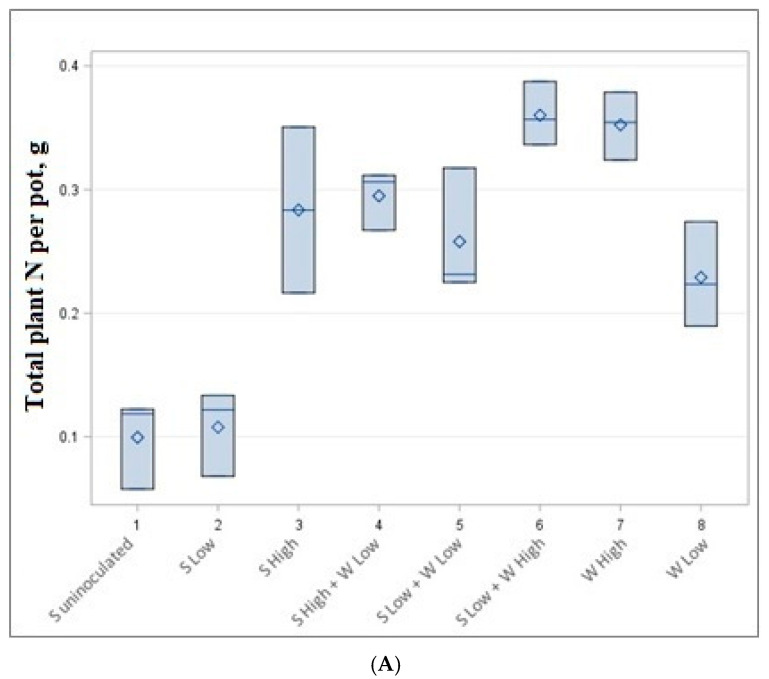

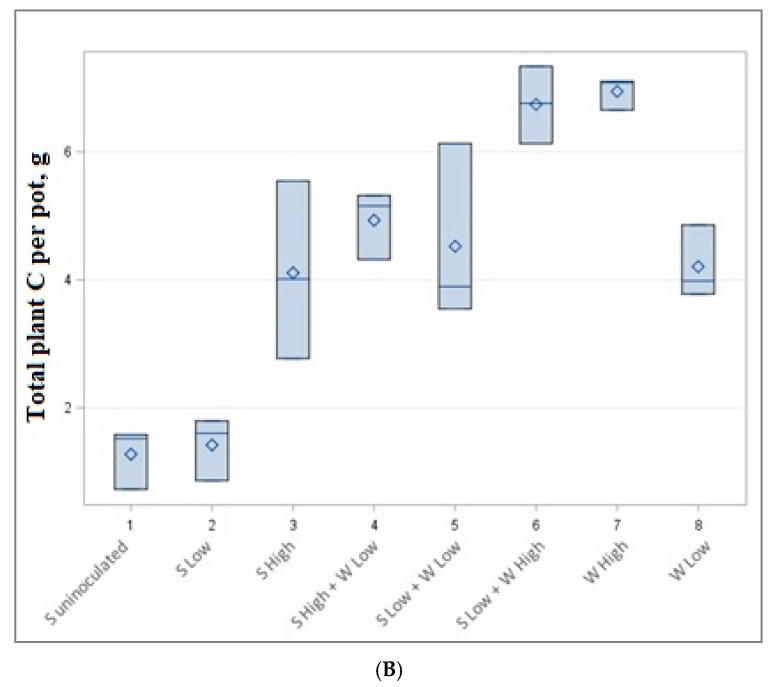
Total nitrogen (**A**) and carbon (**B**) content per pot in g.

The data of accumulated total nitrogen and carbon per pot are presented in Table 4 and Figure 3. The highest but not statistically significantly different carbon (6.74–6.96 g) and nitrogen (0.352–0.360 g) content was accumulated in low soybean density—high wheat density (1 + 8 plants) and high wheat density (8 plants) experiments, although a different number of plants were cultivated. Very similar amounts of C and N (0.258–0.295 g N and 4.11–4.93 g C) were also obtained by growing 3 plants (high-density soybean experiment), 3 + 4 plants (high-density soybean and low-density wheat experiment) and 1 + 8 plants (low-density soybeans low-density spring wheat experiment).

Our experiment shows that soybeans in low quantities produced a lower amount of both carbon and nitrogen per pot. The highest amount of carbon and nitrogen is reached in mixtures of low soybean and high wheat mixtures or high wheat monocultures (Figure 3).

## 3. Discussion

### 3.1. Effect of Intercropping on N Yields and Legume Nitrogen Fixation

Our study using the N isotope dilution method showed that %Ndfa increased significantly in soybean roots from 6% in monocultures to 13–15% in mixtures during the flowering period. This confirms the expected effect of intercropping leading legumes to increased reliance on atmospheric N_2_ [31]. The levels of %Ndfa were relatively low compared to previous studies [32] due to a high soil N fertility [33] and the N added to sustain cereal growth. On a per plant basis, then intercropping reduced soybean N yields to half the sole cropped, whereas N yields per plant in wheat was unaffected by intercropping (Table 3).

Adequate nitrogen accumulation enhances plant vigor, increases crop yields, and improves the quality of agricultural produce. It also plays a vital role in supporting the plant’s metabolic functions and stress responses. In agricultural systems, efficient nitrogen accumulation reduces the need for synthetic fertilizers, promotes sustainable farming practices, and minimizes environmental pollution [34]. In our study, higher N yields per plant were observed for wheat cultivated in monoculture in low densities as compared to the corresponding intercropped wheat (Table 3). This is likely related to the amount of nitrogen that is accessible to individual plants, which would be lower when soybeans also compete for soil nitrogen. On the other hand, the total nitrogen content per pot was the highest in cases of high-density wheat and in mixtures of low soybean and high wheat density (Table 4), which reflects a higher exploration of the soil volume with higher plant density. In terms of carbon accumulation, intercropping can lead to a more efficient use of light, water, and nutrients, promoting greater biomass production [35]. Our study showed that high-density wheat particularly enhanced shoot N and C yields per pot, likely due to increased competition. As a result, it might alleviate microbial community activity in the rhizosphere. Allocated below-ground carbon can be shared with mycorrhizal fungi in exchange for nutrients, and also added into soil as rhizodeposits that potentially increases plant nutrient supply by supporting microbial nutrient mineralization from organic matter [36]. Thus, these interactions can be explored as a potential area for future research.

### 3.2. Carbon Allocation Effects

Resource allocation is often characterized as a trade-off between different functional sinks, such as growth and defense against herbivores and pathogens. Understanding the regulation of carbon allocation is essential to predict plant responses to companion species or environmental changes [37]. In the present study, we ^13^C-labeled the plants in the flowering period to determine whether intercropping and plant density affect plant shoot and root investment. We found that plant density had no effect on C allocation for either wheat or soybean. Intercropping did not affect the C allocation in wheat, but in soybeans, there was a significantly reduced investment in roots for the intercropped plants (Table 2). Plant C allocation would be strongly sink-driven, with photosynthates being preferentially transferred to tissues with the highest demand. That is, under light limitation, plants tend to allocate a higher proportion of assimilated C to above-ground organs, whereas, under reduced nutrient and/or water supply, they invest more C into the root system [38]. Despite of a higher nitrogen fixation activity observed in intercropped soybean, this reduced investment of C in roots at flowering could potentially lead to reduced soybean grain yields. We have previously shown a direct positive link between nodule number and grain yield [39,40], including a strong effect of supporting continued nitrogen fixation activity late in the growth period by additional inoculations. If the intercropping shifts the carbon allocation in favor of above-ground parts, it also tends to affect root health and nodulation in legumes [41,42,43]. If the reduction of root C investments observed here leads to reduced nodule formation and nitrogen fixation, then it could hamper the eventual intercropped soybean yield [40]. Therefore, further investigations are needed to determine yield effects in intercrops and to elucidate whether shifting C investments in the grain legume components offer an explanation for the actual mechanisms of cereal-grain legume interactions.

## 4. Materials and Methods

### 4.1. Experimental Design and Treatments

The experiment was conducted in a greenhouse at Aarhus University, Denmark, AU Viborg, under regulated conditions. Growth conditions in the greenhouse during the entire experiment were controlled: 14/10 h light/dark; photosynthetically active radiation at canopy level: 600 mol m^2^ s^−1^; temperature 19 °C; irrigation of 200 mL each second day per pot.

The soil for the pot experiment was sandy loam (17.3% clay, 30.1% silt, and 52.6% sand). The soil was characterized by a high fertility level with 1.58% total C, 0.15% total N, 140 mg P kg^−1^, 208 mg K kg^−1^, and 100 mg Mg kg^−1^. The soil (11 kg dry weight pot^−1^) was filled in 8.5 l PVC pots (height 31.5 cm, inner diameter 19 cm).

The soybean “Merlin” and spring wheat “Tanium” varieties were sown in pots as monocultures and intercrops with three replications. Each pot contained 4 or 8 plants of wheat, either 1 or 3 soybean plants, or a mixture of both. Treatments were as follows. Monocultures: (1) soybean, not inoculated—control, (2) soybean, low density (1 plant), (3) soybean, high density (3 plants), (4) wheat, low density (4 plants), (5) wheat, high density (8 plants); Mixtures: (6) soybean high density + wheat low density (3 + 4 plants, respectively), (7) soybean low density+ wheat low density (1 + 4 plants, respectively), (8) soybean low density + wheat high density (1 + 8 plants, respectively). Seeds of soybean were inoculated with peat inoculant containing *Bradyrhizobium japonicum* strain AGF78 with 1.5 × 10^9^ colony forming units (CFU) g^−1^.

### 4.2. Isotope Labeling Experiments

The ^15^N isotope dilution method [31] and the ^13^C-CO_2_ pulse labeling technique [44] were used to determine the N fixation activity and plant C allocation, respectively. A complete nutrient solution containing ammonium nitrate (^15^NH_4_—^15^NO_3_, 10 atoms% ^15^N) was used to obtain plants with a high and uniform ^15^N content. The solution was applied to each pot surface just after sowing the plants. Each pot received a total of 5 g N m^−2^. Subsequently, ^15^N enrichment analyses in plants were performed. The crops were pulse-labeled with ^13^CO_2_ three times at 48 h intervals, with each labeling event lasting for a whole photoperiod of 14 h. The plants were harvested for analyses 3 days after the last labeling.

The N_2_-fixation was quantified based on excess atom% ^15^N in soybean and spring wheat growing together as reference plant [45]. The percentage of N derived from the atmosphere (%Ndfa) was calculated using the equation [46]:

%Ndfa = (1 − (excess atom%^15^N legume/excess atom%^15^N reference)) × 100%

where, excess atom% ^15^N was calculated by the difference of ^15^N in soybean grown in unlabeled pots and in the ^15^N–labeled pots; atom% ^15^N reference was calculated in the corresponding species in ^15^N–labeled pots or not inoculated soybean in ^15^N–labeled pots.

The C allocation to above and below ground tissue was determined by tracing ^13^C into shoot and root tissue. The ^13^C enrichment (atom% excess) of each tissue was calculated using equation:

^13^C_atom% excess sample_ = ^13^C_atom% labelled_ − ^13^C_atom% unlabelled_

where ^13^C_atom% labeled_ was generated from labeled samples and ^13^C_atom% unlabeled_ was generated from not labeled samples. ^13^C allocation to the root was counted as the ratio of ^13^C in the shoot and the root.

### 4.3. Biological Parameters of Plants

For the ‘isotope experiment’ in the greenhouse, soybean, and spring wheat were grown for 10 weeks and analyzed during the flowering stage. The plants were further processed individually. Shoots were cut, and roots were separated from the soil by gentle shaking, followed by hand picking and sieving the soil. Roots were washed in water to remove adherent soil. All plant samples were dried at 60 °C for 72 h and weighed for dry matter (DM). Before isotopic and C and N percentage composition analysis, samples were ground to a fine powder using a mill. The samples were weighted, packed into tin capsules, and measured for total N concentration, atom% ^15^N, total C concentration, and atom% ^13^C content at UC Davis Stable Isotope Facility, University of California, USA, on an ANCA-SL Elemental Analyser coupled to a 20–20 Mass Spectrometer using the Dumas dry-combustion method.

### 4.4. Data Analysis

All statistical analyses were performed with SAS software version 9.4 (SAS Institute Inc., Copyright © 2002–2010). Homogeneity and normality were verified using Bartlett’s test. The experimental data were analyzed by one-way analysis of variance (ANOVA), and mean comparisons between treatments were performed using Tukey’s mean separation test. The smallest significant difference, R_05_, was calculated using a probability level of *p* < 0.05. Where required, repeated measures analysis was used to test for changing effects over time. Interrelationships among the data were estimated separately for each experiment.

## 5. Conclusions

The intercropping with spring wheat had no effect on soybean height, shoots, and root biomass but significantly increased soybean atmospheric nitrogen fixation and reduced belowground C allocation in soybeans. Our study using the N isotope dilution method showed that %Ndfa increased significantly in soybean roots from 6% in monocultures to 13–15% in mixtures during the flowering period. This confirms the expected effect of intercropping leading legumes to increased reliance on atmospheric N_2_.

Spring wheat biomass was unaffected by intercropping with soybean but showed a higher accumulation of C and N with increasing plant density in both monoculture and mixtures.

We specifically investigated C allocation around soybean flowering and found a clear difference between the two species, where wheat was unaffected by intercropping and soybean reduced root C investments. Such a reduced below ground C investment could lead to reduced soybean yield, if the total C investment in the apparatus for nitrogen fixation would also be reduced. Therefore, a deeper understanding of plant responses to companion species is crucial for optimizing intercropping systems and ensuring the productivity benefits of such cultivation methods.

## Figures and Tables

**Table 1:** Average values of plant height, biomass, nodule number per plant, and C:N ratios in the shoots and roots of soybean and spring wheat grown as monocultures or intercrops at various plant densities (numbers in brackets).

Cropping System	Height, cm	Nodule Plant^−1^	Shoot Weight, g Plant^−1^ DM	Root Weight, g Plant^−1^ DM	C:N Shoots	C:N Roots
**Soybean (S)**						
**Monoculture:**						
S uninoculated	27.2 a	0.0 a	2.41 a	0.68 ab	13.4 a	10.8 b
S Low (1)	29.3 a	3.0 a	2.68 a	0.73 a	13.7 a	11.2 b
S High (3)	28.1 a	6.2 a	2.62 a	0.66 ab	14.8 a	12.3 b
**Mixtures S/W**						
High/Low (3/4)	30.4 a	12.3 a	1.52 a	0.34 b	15.9 a	16.0 a
Low/Low (1/4)	34.0 a	5.7 a	2.01 a	0.46 ab	14.9 a	16.7 a
Low/High (1/8)	32.7 a	13.7 a	1.60 a	0.37 ab	17.0 a	17.9 a
**Wheat (W)**						
**Monoculture:**						
W Low (4)	50.3 a	NA	2.13 a	0.49 a	18.3 ab	19.8 b
W High (8)	45.0 b	NA	1.78 ab	0.34 b	19.5 a	22.1 ab
**Mixtures S/W**						
High/Low (3/4)	43.2 b	NA	1.27 b	0.22 b	17.2 b	20.8 ab
Low/Low (1/4)	44.0 b	NA	1.65 ab	0.28 b	17.8 ab	21.4 ab
Low/High (1/8)	43.6 b	NA	1.65 ab	0.24 b	18.3 ab	24.9 a

Means followed by the same letters in the same column and section do not differ from one another (*p* ≤ 0.05). Notes: S—soybean, W—spring wheat, High/Low—high and low plant densities, %Ndfa—% of nitrogen derived from the atmosphere, NA—not applicable.

**Table 2:** Nitrogen content and Ndfa, as well as ^13^C isotope allocation in the shoot and root of soybean and spring wheat, cultivated as monocultures or intercrops at various plant densities (numbers in brackets).

Cropping System	N Shoots, %	N Roots, %	Ndfa Roots, %	Ndfa Shoots, %	C Shoots, %	C Roots, %	^13^C Roots, Atom% Excess	^13^C Shoots, Atom% Excess	^13^C Shoot-to-Root Allocation, %
				**Soybean (S)**					
**Monoculture:**									
S uninoculated	3.14 a	3.59 a	0.0 c	0.0 c	42.2 b	38.8 bc	2.07 a	2.79 a	42.2 a
S Low (1)	3.10 a	3.50 a	6.0 b	5.1 ab	42.6 ab	39.0 bc	1.96 a	2.82 a	40.9 a
S High (3)	2.91 a	3.12 a	6.0 b	2.5 bc	42.8 ab	38.4 c	1.37 b	1.95 b	41.5 a
**Mixtures S/W**									
High/Low (3/4)	2.73 a	2.64 b	12.8 a	4.6 abc	43.3 ab	42.1 a	1.01 bc	1.82 b	35.8 b
Low/Low (1/4)	3.06 a	2.49 b	12.2 a	2.9 bc	44.5 a	40.8 ab	0.94 bc	1.85 b	33.2 b
Low/High (1/8)	2.63 a	2.39 b	15.3 a	9.2 a	43.7 ab	42.2 a	0.65 c	1.27 c	33.9 b
				**Wheat (W)**					
**Monoculture:**									
W Low (4)	2.33 bc	1.56 ab	NA	NA	42.5 b	30.9 b	1.43 a	1.91 a	42.8 a
W High (8)	2.19 c	1.49 b	NA	NA	42.6 b	32.7 b	1.02 b	1.39 b	42.4 a
**Mixtures S/W**									
High/Low (3/4)	2.52 ab	1.88 a	NA	NA	43.2 ab	39.0 a	1.20 ab	1.77 a	40.2 a
Low/Low (1/4)	2.55 a	1.85 ab	NA	NA	45.4 a	38.6 a	1.27 ab	1.75 a	43.5 a
Low/High (1/8)	2.39 ab	1.55 ab	NA	NA	43.9 ab	38.4 a	0.99 b	1.24 b	44.0 a

Means followed by the same letters in the same column and section do not differ from one another (*p* ≤ 0.05). Notes: S—soybean, W—spring wheat, High/low—high and low plant densities, %Ndfa—% of nitrogen derived from the atmosphere, NA—not applicable.

**Table 3:** Total accumulated nitrogen and carbon (g) in the shoots and roots of the individual soybean and spring wheat plants, cultivated as monocultures or intercrops at various plant densities (numbers in brackets).

Cropping System	Total N Per Shoot	Total N Per Root	Total N Per Plant	Total C Per Shoot	Total C Per Root	Total C Per Plant
**Soybean (S)**						
**Monoculture:**						
S uninoculated	0.076 ab	0.024 a	0.100 a	1.05 a	0.26 a	1.28 a
S Low (1)	0.082 a	0.025 a	0.108 a	1.14 a	0.28 a	1.42 a
S High (3)	0.074 ab	0.020 ab	0.095 a	1.12 a	0.25 a	1.37 a
**Mixtures S/W**						
High/Low (3/4)	0.041 b	0.009 c	0.050 b	0.66 a	0.14 a	0.80 a
Low/Low (1/4)	0.060 ab	0.011 bc	0.071 ab	0.90 a	0.19 a	1.09 a
Low/High (1/8)	0.041 b	0.009 c	0.050 b	0.70 a	0.16 a	0.86 a
**Wheat (W)**						
**Monoculture:**						
W Low (4)	0.050 a	0.0077 a	0.057 a	0.90 a	0.15 a	1.05 a
W High (8)	0.039 ab	0.0051 b	0.044 ab	0.76 ab	0.11 ab	0.87 ab
**Mixtures S/W**						
High/Low (3/4)	0.032 b	0.0041 b	0.036 b	0.55 b	0.08 b	0.63 b
Low/Low (1/4)	0.042 ab	0.0050 b	0.047 ab	0.75 ab	0.11 ab	0.86 ab
Low/High (1/8)	0.035 b	0.0038 b	0.039 b	0.64 b	0.09 b	0.74 b

Means followed by the same letters in the same column and section do not differ from one another (*p* ≤ 0.05). Notes: S—soybean, W—spring wheat, High/Low—high and low plant densities.

**Table 4:** Total accumulated nitrogen and carbon in the shoot and root of soybean and spring wheat, calculated for the pot at various plant densities (numbers in brackets).

Cropping System	Total Shoot N Per Pot	Total Root N Per Pot	Total Plant N Per Pot	Total Shoot C Per Pot	Total Root C Per Pot	Total Plant C Per Pot
**Soybean (S)**						
**Monoculture:**						
S uninoculated	0.076 c	0.024 c	0.100 c	1.05 c	0.26 c	1.28 c
S Low (1)	0.082 c	0.025 c	0.108 c	1.14 c	0.28 c	1.42 c
S High (3)	0.222 b	0.060 a	0.285 ab	3.36 b	0.75 ab	4.11 b
**Mixtures S/W**						
High/Low (3/4)	0.252 b	0.0430 b	0.295 ab	4.17 b	0.77 ab	4.93 b
Low/Low (1/4)	0.227 b	0.0311 bc	0.258 b	3.90 b	0.63 ab	4.53 b
Low/High (1/8)	0.322 a	0.0386 bc	0.360 a	5.84 a	0.90 a	6.74 a
**Monoculture:**			**Wheat (W)**			
W High (8)	0.312 a	0.0408 bc	0.352 a	6.08 a	0.88 a	6.96 a
W Low (4)	0.200 b	0.0308 bc	0.228 b	3.60 b	0.60 b	4.20 b

Means followed by the same letters in the same column and section do not differ from one another (*p* ≤ 0.05). Notes: S—soybean, W—spring wheat, High/Low—high and low plant densities, %Ndfa—% of nitrogen derived from the atmosphere, NA—not applicable.

## Data Availability

The original contributions presented in this study are included in the article. Further inquiries can be directed to the corresponding author.
